# Endothelial Progenitor Cell-Derived Microvesicles Promote Angiogenesis in Rat Brain Microvascular Endothelial Cells *In vitro*

**DOI:** 10.3389/fncel.2021.638351

**Published:** 2021-02-18

**Authors:** Wen Zeng, Qiaoling Lei, Jiao Ma, Shuqiang Gao, Rong Ju

**Affiliations:** Department of Neonatology, Chengdu Women’s and Children’s Central Hospital, School of Medicine, University of Electronic Science and Technology of China, Chengdu, China

**Keywords:** microvesicle, angiogenesis, endothelial progenitor cell, brain microvascular endothelial cell, proliferation, migration

## Abstract

Brain microvascular endothelial cells (BMECs) are a major component of the blood-brain barrier that maintains brain homeostasis. Preserving and restoring the normal biological functions of BMECs can reverse or reduce brain injury. Endothelial progenitor cells (EPCs) may promote brain vascular remodeling and restore normal endothelial function. As a novel vehicle for cell-cell communication, microvesicles (MVs) have varied biological functions. The present study investigated the biological effects of EPC-derived MVs (EPC-MVs) on BMECs *in vitro*. We isolated MVs from the supernatant of EPCs in a serum-depleted medium. BMECs were cultured alone or in the presence of EPC-MVs. BMEC viability and proliferation were evaluated with the Cell Counting Kit-8 and by flow cytometry, and the proangiogenic effect of EPC-MVs on BMECs was assessed with the transwell migration, wound healing, and tube formation assays. Our results showed that EPC-derived MVs labeled with DiI were internalized by cultured BMECs; this enhanced BMEC viability and promoted their proliferation. EPC-MVs also stimulated migration and tube formation in BMECs. These results demonstrate that EPC-derived MVs exert a proangiogenic effect on BMECs, which has potential applications in cell-free therapy for brain injury.

## Introduction

Endothelial cell (EC) dysfunction contributes to brain injury in many pathologic conditions (Grammas et al., 2011). Brain angiogenesis can restore the function of the blood-brain barrier (BBB) and promote functional recovery from brain injury (Persidsky et al., 2006; Freitas-Andrade et al., 2020). Brain microvascular (BM) ECs, a major component of the BBB that maintains brain homeostasis, activates angiogenesis (Ruck et al., 2014; Huang et al., 2019), and is involved in vascular sprouting and tube formation through interactions with the extracellular matrix (ECM).

Microvesicles (MVs) are small membrane particles 0.1–1 μm in size that are released by various cell types including endothelial progenitor cells (EPCs) and deliver proteins, as well as mRNA and micro (mi)RNA, to recipient cells (Deregibus et al., 2007; Cantaluppi et al., 2012a; van Niel et al., 2018; Shu et al., 2019), presenting a novel form of cell-cell communication. This biological function of MVs depends on the cell from which they originate. As EPCs are important for endothelial function (Hristov and Weber, 2004; Urbich and Dimmeler, 2004), we speculated that MVs derived from EPCs (EPC-MVs) can promote angiogenesis in the brain. We tested this hypothesis in the present study by evaluating the effects of EPC-MVs on the proliferation, migration, and angiogenic potential of cultured BMECs.

## Materials and Methods

### Culture and Characterization of EPCs

Mononuclear cells were isolated from the spleens of male Sprague-Dawley rats (*n* = 2, 12 weeks old; Third Military Medical University, Chongqing, China) and grown in EC basal medium 2 (cat. no. CC-3156; Lonza, Walkersville, MD, USA), supplemented with 5% fetal bovine serum (cat. no. 10099141C; Thermo Fisher Scientific, Waltham, MA, USA) at 37°C and 5% CO_2_. After 3 days, nonadherent cells were removed by rinsing with phosphate-buffered saline (PBS). Thereafter, the culture medium was changed every 2 days, and cells were passaged at a ratio of 1:2 when they reached 80% confluence. Passage 3 EPCs were collected for experiments. All procedures conformed to local regulations for laboratory animal use and were approved by Chengdu Women’s and Children’s Central Hospital (Sichuan, China).

EPCs were characterized by flow cytometry analysis. Cultured cells were trypsinized and resuspended in PBS containing 1% bovine serum albumin (cat. no. 30063481; Thermo Fisher Scientific, Waltham, MA, USA). Cell suspensions were incubated at room temperature for 30 min in the dark with fluorescein isothiocyanate (FITC)-conjugated antibodies against a cluster of differentiation (CD)133 (1:100; cat. no. sc-365537), CD34 (1:50; cat. no. sc-7324), and von Willebrand factor (vWF; 1:200; cat. no. sc-365712; all from Santa Cruz Biotechnology, Dallas, TX, USA); and an Alexa Fluor 647-conjugated antibody against vascular endothelial growth factor receptor (VEGFR)-2 (1:50; cat. no. 89B3A5; BioLegend, San Diego, CA, USA). Isotype-matched IgG served as negative controls. Labeled cells were resuspended in PBS and analyzed by flow cytometry (BD Biosciences, Franklin Lakes, NJ, USA).

### Primary Culture of BMECs

BMECs were prepared from Sprague–Dawley rats (*n* = 8, 3 weeks old). Cortical tissue was cleaned of meninges and superficial blood vessels, minced into small pieces (~1–2 mm^3^), homogenized and centrifuged. Microvessel-containing pellets were digested with 0.1% collagenase II (cat. no. C8150; Solarbio, Beijing, China) and DNase at 37°C for 1.5 h and BMECs were concentrated by centrifugation. The cells were resuspended in Dulbecco’s Modified Eagle’s Medium (DMEM)-F12 (cat. no. 11320033; Thermo Fisher Scientific, Waltham, MA, USA) and plated on culture dishes coated with the extracellular matrix. Cells were grown in DMEM-F12 containing 10% fetal bovine serum, 4 mmol/l glutamine (cat. no. 25030081), 1 ng/ml basic fibroblast growth factor (cat. no. 13256-029), and 100 U/ml streptomycin and penicillin (cat. no. 15070063; all from Thermo Fisher Scientific, Waltham, MA, USA); and 100 mg/l heparin (cat. no. H3149; Sigma–Aldrich, St. Louis, MO, USA) at 37°C and 5% CO_2_. Only primary BMECs were used for experiments and were cultured alone or in the presence of EPC-MVs (10 μg/ml).

### Mycoplasma Test

Mycoplasma testing was performed with the MycoAlert Mycoplasma Detection Kit (cat. no. LT07-118; Lonza) according to the manufacturer’s protocol.

### Collection and Characterization of EPC-MVs

EPCs at 80% confluence were cultured in serum-free medium for 24 h; the culture medium was collected and centrifuged at 4°C (300× *g* for 5 min followed by 2,000× *g* for 15 min). The supernatant was ultracentrifuged at 100,000× *g* (Optima L-80; Beckman Coulter, Brea, CA, USA) for 2 h at 4°C, and resuspended in PBS followed by ultracentrifugation at 100,000× *g* for 1 h at 4°C. EPC-MVs were characterized by Transmission electron microscopy (TEM) and flow cytometry analysis. For TEM, MVs were fixed in Karnovsky fixative, dehydrated in alcohol, dried on a glass surface, and sputter-coated with gold. The specimens were visualized using an FEI Tecnai-10 transmission electron microscope (Philips, Amsterdam, The Netherlands). For flow cytometry analysis, EPC-MVs were resuspended and incubated for 30 min at 4°C in the dark with phycoerythrin (PE)-conjugated antibodies against CD34 (1:50; cat. no. sc-7324) and FITC-conjugated antibodies against annexin V (1:100; cat. no. sc-74438; all from Santa Cruz Biotechnology). An isotype-matched (IgG) nonspecific antibody served as a negative control. After incubation, labeled MVs were washed with PBS three times, resuspended with 70 μl of PBS, and analyzed by flow cytometry (BD Biosciences).

### DiI Labeling of EPC-MVs

EPC-MVs were labeled with 10 μM DiI (cat. no. 42364; Sigma–Aldrich) in PBS for 10 min at room temperature; the labeling was terminated by adding the same volume of fetal bovine serum. The labeled EPC-MVs were added to BMECs, which were cultured at 37°C and 5% CO_2_ for 24 h. Cell nuclei were stained with DAPI (cat. no. MBD0015; Sigma–Aldrich), and the interaction between EPC-MVs and BMECs was observed with a fluorescence microscope (Nikon, Tokyo, Japan).

### Cell Counting Kit-8 (CCK-8) Assay

Cell viability was assessed with the CCK-8 assay (cat. no. CK04; Dojindo, Osaka, Japan) according to the manufacturer’s instructions. BMECs were seeded in 96-well plates (5 × 10^4^ cells/well) and 10 μl CCK-8 solution was added to each well. The optical density was measured with a microplate reader (Bio-Rad, Hercules, CA, USA) at a wavelength of 450 nm; the value was used to calculate cell viability by setting the control as 100%.

### Cell Cycle Analysis by Flow Cytometry

Cells were harvested by trypsinization and washed twice with ice-cold PBS, then resuspended and mixed with a buffer containing trypsin inhibitor (cat. no. T8031) and RNase (cat. no. R1030; both from Solarbio) at room temperature for 10 min. Propidium iodide (50 μg/ml; cat. no. P4170; Sigma–Aldrich) was added and cells were incubated at 4°C for 10 min in the dark. The cell suspension was filtered through a 200-mesh sieve and cell cycle distribution was analyzed with a FACScan flow cytometer (BD Biosciences) at excitation/emission wavelengths of 488/630 nm. The percentage of cells in each phase of the cell cycle calculated.

### Transwell Migration Assay

Cell migration was evaluated using a 24-well plate containing transwell inserts with 8.0 μm pores (Corning, Corning, NY, USA). BMECs (2 × 10^4^ cells/well) were seeded in the upper chambers in DMEM, while DMEM supplemented with 10% fetal bovine serum was added to the lower chamber. Migration was assessed after 12 h culture. Cells adhering on the topside of the transwell insert were removed with a cell swab; those attached to the underside were fixed with 70% ethanol and stained with 0.1% crystal violet, and the number of cells was counted under light microscopy.

### Wound Healing Assay

BMECs were seeded in 6-well plates; when they reached 80% confluence, the culture medium was removed by aspiration and a 200-μl pipette tip was used to scratch the cell monolayer. The plates were washed three times with PBS to remove nonadherent cells, and a serum-free medium was added. Images of the cells were obtained by phase-contrast light microscopy at 0, 6, 12, and 24 h after creating the scratch wound.

### Tube Formation Assay

The ability of BMECs to form capillary tubes was evaluated using Matrigel matrix (cat. no. 354234; BD Biosciences) according to the manufacturer’s instructions. BMECs were seeded (1 × 10^4^ cells/well) on the surface of the solidified Matrigel matrix and incubated for 24 h at 37°C, and capillary tube formation was evaluated with an inverted light microscope. The number of tubes was counted using ImageJ image software (National Institutes of Health, Bethesda, MD, USA).

### Statistical Analysis

Data are expressed as the mean ± SD and were analyzed using SPSS 22.0 software (SPSS Inc. Chicago, IL, USA). The Student’s *t*-test was used for comparisons between two groups and analysis of variance followed by a *post hoc* Bonferroni correction was used for multigroup comparisons, with *p* < 0.05 set as the threshold for statistical significance.

## Results

### Internalization of EPC-MVs by Co-cultured BMECs

**Figure 1:**
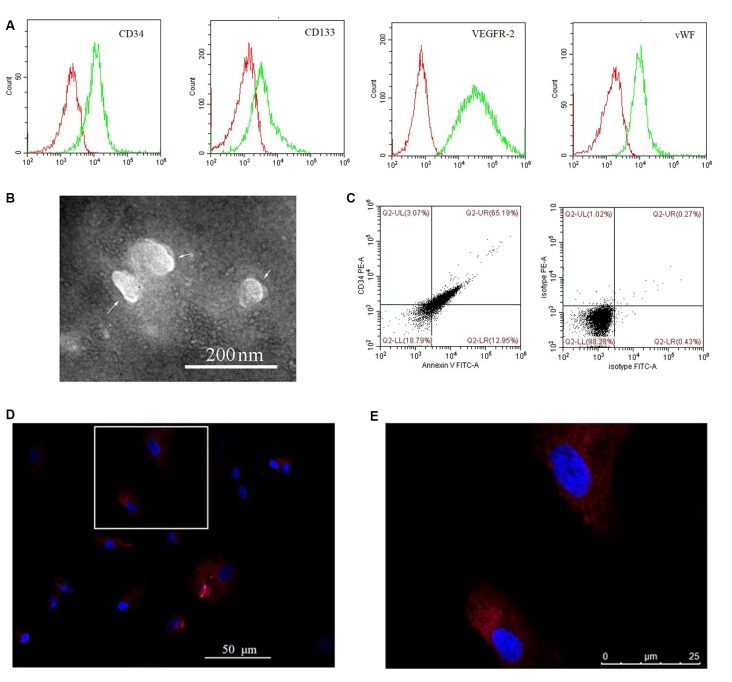
Internalization of endothelial progenitor cell-microvesicles (EPC-MVs) by brain microvascular endothelial cells (BMECs). **(A)** Representative flow cytometry plots of CD34, CD133, VEGFR-2, and vWF levels in cultured EPCs. Red and green curves represent the isotype controls and markers, respectively. **(B)** EPC-MV morphology observed by transmission electron microscopy (TEM). Arrows indicate EPC-MVs. Scale bar: 200 nm. **(C)** EPC-MVs are characterized by flow cytometry. Left: Annexin V and CD34 on EPC-MVs. Right: Isotype controls for EPC-MVs. **(D,E)** Internalization of EPC-MVs by BMECs detected by fluorescence microscopy. EPC-MVs were labeled with DiI (red) and cell nuclei were stained with DAPI (blue). The square area in panel **(D)** is shown at higher magnification in panel **(E)**. Scale bars: 50 μm **(D)**, 25 μm **(E)**.

Cultured EPCs and BMECs tested negative for mycoplasma (data not shown). Cultured EPCs were characterized by flow cytometry as positive for CD34, CD133, VEGFR-2, and vWF (Figure 1A), thus confirming their identity. EPC-MVs were collected from cultures of EPCs. The morphology of EPC-MVs was examined by TEM (Figure 1B). The flow cytometry results revealed that EPC-MVs had externalized phosphatidylserine, which was detected by annexin V staining, and expressed the EPC marker CD34 (Figure 1C). BMECs were incubated with DiI-labeled EPC-MVs for 24 h, DiI fluorescence was detected in the cytoplasm, suggesting that the MVs were internalized by the cells (Figures 1D,E).

### EPC-MVs Enhance BMEC Viability and Proliferation

**Figure 2:**
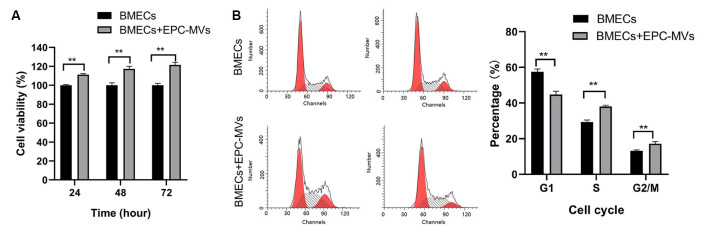
Effects of EPC-MVs on BMEC viability and proliferation. **(A)** Effects of EPC-MVs on BMEC viability, as determined with the CCK-8 assay. The proportion of viable cells was higher in the BMECs + EPC-MV group compared to the BMEC only group (*p* < 0.01). **(B)** Effects of EPC-MVs on BMEC cell cycle as determined by flow cytometry. Cell cycle entry at 24 h was increased in the BMEC + EPC-MV group, as evidenced by the larger S- and G2/M-phase fractions in the DNA histogram (*p* < 0.01). Results are expressed as mean ± SD of at least three experiments. **p < 0.01.

We evaluated the effects of EPC-MVs on BMEC viability and proliferation with the CCK-8 assay. Incubation with EPC-MVs increased the proliferation rate of BMECs (Figure 2A). To investigate whether EPC-MVs promote the proliferation of BMECs, we analyzed cell cycle distribution by flow cytometry. The proportion of cells in S and G2/M phase were higher in BMECs cultured with EPC-MVs than in those cultured alone (S phase: 29.35% ± 1.15% vs. 38.05% ± 0.71%; G2/M phase: 13.15% ± 0.48% vs. 17.16% ± 1.15%; both *p* < 0.01), whereas the opposite trend was observed for G1 phase (57.50% ± 1.59% vs. 44.79% ± 1.82%; *p* < 0.01; Figure 2B). These results indicate that EPC-MVs induced cell cycle entry by BMECs.

### EPC-MVs Promote BMEC Migration

**Figure 3:**
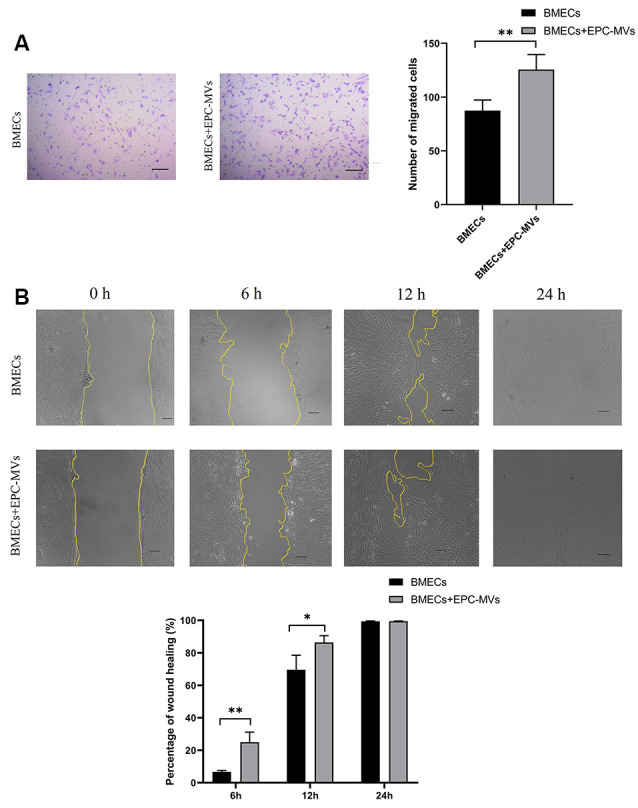
Effects of EPC-MVs on BMEC migration. **(A)** Left: representative images from the transwell migration assay. Cells were stained with crystal violet. Right: quantitative analysis of results from the transwell migration assay. Scale bar: 10 μm. **(B)** Upper panel: phase-contrast micrographs of BMECs in the wound-healing assay at 0, 6, 12, and 24 h. Yellow lines delineate the wound edges. Scale bar: 10 μm. Lower panel: quantitative analysis of results from the wound healing assay. Results are expressed as mean ± SD of at least three experiments. *p < 0.05, **p < 0.01.

BMECs migration was evaluated with the transwell migration and wound healing assays. Cells that migrated across the transwell membrane were stained with crystal violet and counted under light microscopy. EPC-MVs markedly enhanced the migration of BMECs compared to cells cultured alone (87.40 ± 9.91 vs. 125.60 ± 13.99; *p* < 0.01; Figure 3A). In the wound healing assay, the wound area was significantly smaller in BMECs treated with EPC-MVs than in control BMECs cultures at 6 h (6.67% ± 0.91% vs. 24.99% ± 6.19%, *p* < 0.01) and 12 h (69.56% ± 8.99% vs. 86.43% ± 4.18%, *p* < 0.05; Figure 3B), indicating that EPC-MVs accelerated wound healing by promoting BMEC migration. However, there was no significant difference in the wound area at 24 h (99.46% ± 0.23% vs. 99.69% ± 0.16%, *p* > 0.05).

### EPC-MVs Induce Tube Formation in BMEC

**Figure 4:**
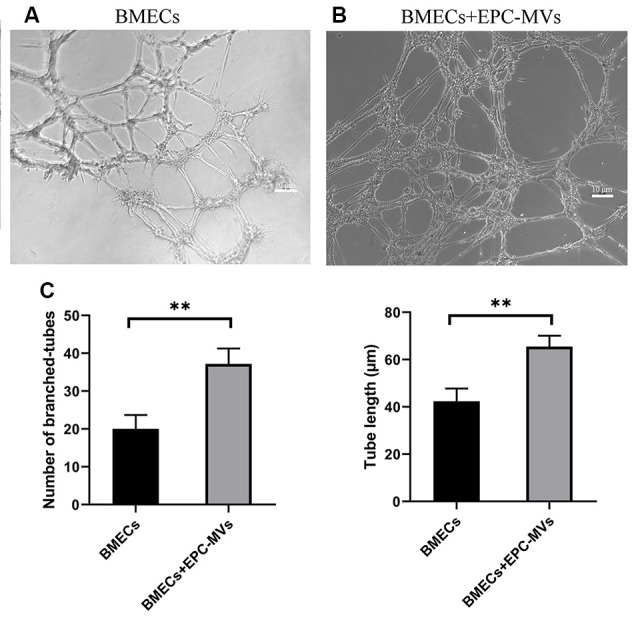
Effects of EPC-MVs on tube formation by BMECs. **(A,B)** Representative images of tube formation by BMECs. Scale bar: 10 μm. **(C)** Quantitative analysis of branched tube formation and tube length. Results are expressed as mean ± SD of at least three experiments. **p < 0.01.

To investigate the angiogenic potential of EPC-MVs, we used collagen gels to examine tube formation by BMECs cultured in the presence of the MVs (Figures 4A,B). Tube formation was increased in cells incubated with EPC-MVs compared to control cultures (20.04 ± 3.63 vs. 37.2 ± 4.08; *p* < 0.01), and the length of tubes that were formed was also greater (42.38 ± 5.39 vs. 65.49 ± 4.60; *p* < 0.01; Figure 4C), providing evidence that EPC-MVs can induce vessel formation by BMECs.

## Discussion

BMECs interact with astrocytes and pericytes to form the BBB (Nakagawa et al., 2007), which protects neurons from factors present in the systemic circulation and regulates the internal milieu of the central nervous system (CNS), thus ensuring normal neuronal function (Zhao et al., 2015; Sweeney et al., 2018). Upon brain injury, energy balance, glia–neuron interactions, and the BBB are disrupted, leading to synaptic dysfunction and loss of neuronal connectivity (Sweeney et al., 2018; Bordone et al., 2019). This is observed in various types of brain injuries including ischemic stroke, intracranial hemorrhage, and traumatic brain injury (TBI; Prakash and Carmichael, 2015; Zhu et al., 2018; Yang et al., 2019; Wu et al., 2020). Given the essential role of the BBB in maintaining a stable CNS microenvironment (Sá-Pereira et al., 2012), therapeutic strategies that promote BMEC functions may be effective in repairing brain injury.

Vascular remodeling following stroke and TBI involves angiogenesis (Liu et al., 2014; Russo et al., 2018), in which new vessels form from the pre-existing vascular network through the proliferation and migration of ECs. Angiogenesis promotes tissue repair and can preserve neurologic function by improving tissue perfusion (Yin et al., 2015; Li et al., 2016; Fan et al., 2018) and restoring BBB integrity following cerebral ischemia (Abdulkadir et al., 2020). EPCs promote brain vascular remodeling and restore normal endothelial function by releasing growth factors and differentiating into mature ECs (Hristov and Weber, 2004). Thus, EPCs have therapeutic potential for the treatment of brain injury. Indeed, increasing the number of circulating EPCs promoted the recovery of neurologic function in animal models of TBI (Wang et al., 2015), whereas inhibiting EPC motility decreased angiogenesis around the lesion and prevented functional restoration after TBI (Zhang et al., 2019). As EPCs maintain endothelial integrity and promote vascular growth and neovascularization, their transplantation is a promising therapeutic approach for brain injury, although the safety and effectiveness of such treatment have yet to be established (Kaneko et al., 2012). A cell-free strategy has been proposed as an alternative to cell transplantation based on the observation that paracrine signals from EPCs are involved in tissue repair. For example, factors secreted by EPCs were shown to enhance EC survival, morphogenesis, and migration as well as tissue revascularization and functional recovery (Di Santo et al., 2014).

MVs are extracellular vesicles released by various cell types including EPCs that play a critical role in cell-cell communication under normal physiologic as well as pathologic conditions. MVs modulate multiple processes including vascular function, angiogenesis, and cell proliferation. EPC-MVs were shown to protect cardiomyocytes against angiotensin-II-induced hypertrophy and apoptosis (Gu et al., 2014); they also induced neoangiogenesis and enhanced tissue recovery in a mouse model of hindlimb ischemia (Ranghino et al., 2012), and protected the kidney from an acute ischemic injury, which involved the modulation of angiogenesis (Cantaluppi et al., 2012b). However, there have been no studies on the effects of EPC-MVs in BMECs. In the present study, we investigated whether substances secreted by EPCs *via* MVs can promote angiogenic functions in BMECs. We found that MVs isolated from the EPC culture medium stimulated proliferation, migration, and tube formation in BMECs, which are important aspects of angiogenesis. The biological functions of MVs are closely related to their contents. MVs are a major vehicle for circulating miRNA (Shu et al., 2019). We previously reported that RNase pretreatment reduced the proliferative effect of EPC-MVs containing miR-210 (Zeng et al., 2020). Similarly, RNase inactivation of EPC-MVs containing the miRNAs miRNA-126 and miRNA-296 inhibited their proangiogenic effect (Ranghino et al., 2012), which was corroborated by another study (Cantaluppi et al., 2012b). Thus, EPC-MVs exert some of their biological functions through the shuttling of miRNAs and represent a novel vehicle for cell-free therapies (Di Santo et al., 2009; Chen et al., 2014).

Glial cells—especially astrocytes—are essential for maintaining the integrity of the BBB. The channel protein aquaporin (AQP)4 expressed in astrocytes mediates water flux across the BBB, which is relevant to the treatment of brain edema (Kitchen et al., 2020). Pericytes are not only required for BBB formation during development but also contribute to its functions in vesicle trafficking and vascular permeability *via* regulation of tight junctions (Daneman et al., 2010). Our data indicate that BMECs exposed to EPC-MVs also have therapeutic potential for the treatment of neurologic diseases associated with BBB disruption. However, our experiments used 2-dimensional monocultures of BMECs, which do not fully recapitulate the cytoarchitecture and synaptic connectivity of the brain, thus limiting their utility for disease modeling or drug screening (Centeno et al., 2018). Three-dimensional models more closely reflect the structural and functional complexities of the brain (Lee et al., 2017; Chang et al., 2020). An open microfluidic brain microvessel-on-a-chip model of the BBB was recently developed (Salman et al., 2020). Further studies on the role of EPC-MVs in modulating brain angiogenesis and maintaining the BBB using 3-dimensional models are needed to develop clinically effective cell-free therapies for brain injury.

## Data Availability Statement

The original contributions presented in the study are included in the article, further inquiries can be directed to the corresponding author.

## Ethics Statement

The animal study was reviewed and approved by Chengdu Women’s and Children’s Central Hospital.

## Author Contributions

WZ designed the study, performed the experiments, analyzed the data, and wrote the manuscript. QL performed cell culture, prepared MVs, and carried out the TEM analysis. JM performed flow cytometry, and the transwell and wound healing assays. SG performed cell culture, and the CCK-8 and tube formation assays. RJ collected and analyzed the data and revised the manuscript. All authors contributed to the article and approved the submitted version.

## Conflict of Interest

The authors declare that the research was conducted in the absence of any commercial or financial relationships that could be construed as a potential conflict of interest.
